# Assessing the Readiness of Local Vaccine Manufacturing in African Countries: Protocol for a Scoping Review

**DOI:** 10.2196/81231

**Published:** 2025-12-23

**Authors:** Uchenna Anderson Amaechi, Chukwudi Arnest Nnaji, Kelechi Julian Uzor, Justice Nonvignon, Nicolas Ray

**Affiliations:** 1 Institute of Global Health Faculty of Medicine, University of Geneva Geneva Switzerland; 2 Malaria Consortium London United Kingdom; 3 Harvard University Cambridge, MA United States; 4 Management Sciences for Health Arlington, VA United States; 5 University of Ghana Accra, Greater Accra Ghana

**Keywords:** vaccines, manufacturing, readiness, Africa, frameworks, indicators, scoping review, PRISMA-ScR, Preferred Reporting Items for Systematic Reviews and Meta-Analyses extension for Scoping Reviews, policy, capacity building

## Abstract

**Background:**

Although Africa experiences the highest burden of infectious diseases, the continent currently produces less than 1% of its vaccine needs. In 2021, the African Union set a target to locally produce at least 60% of the continent’s vaccine needs by 2040. However, at the time of developing this scoping review protocol, there is no consolidated, evidence-based framework for assessing national or regional “readiness” to establish or scale vaccine production.

**Objective:**

This protocol aims to describe a methodological approach that will be used to review existing literature to identify, map, and synthesize the existing evidence on all relevant frameworks, indicator sets, and policy documents (global or national) developed pre- and post–COVID-19 pandemic (January 1, 2010, to December 31, 2025) that addresses readiness for local human vaccine manufacturing with focus on African countries.

**Methods:**

This scoping review will be conducted and reported in accordance with the PRISMA-ScR (Preferred Reporting Items for Systematic Reviews and Meta-Analyses extension for Scoping Reviews) guidelines, following the 9-step framework outlined in the Arksey and O’Malley methodology and further informed by guidance from the Joanna Briggs Institute. We will search MEDLINE (PubMed), Scopus, Web of Science, Africa-focused databases (eg, Africa-Wide Information, African Index Medicus, and African Journals Online), and gray literature. Eligibility criteria will follow Population, Concept, and Context guidelines (Population: 55 African Union member states; Concept: readiness frameworks, indices, indicators, and policies for human vaccine manufacturing; Context: African national or regional initiatives or global frameworks applied to Africa). Materials in English, French, Portuguese, or Arabic will be included. Publication types will be limited to frameworks, policies, guidance, and reports. Two reviewers will perform calibrated dual screening (Cohen κ) and standardized data charting. We will create an evidence map and inductive thematic synthesis using a vaccine-specific Political, Economic, Social, Technological, Legal, Environmental, plus Market taxonomy. Consistent with the guidance by the Joanna Briggs Institute, critical appraisal will not be performed. An optional expert consultation will help identify missed sources and validate domains.

**Results:**

Ethics approval for the expert consultation component was obtained from the University of Geneva Research Ethics Committee (application submitted May 21, 2025; approval September 9, 2025). The initial search strategy has been finalized, and pilot searches were completed (May-August 2025). The screening calibration is planned; dual-review title and abstract screening begins in December 2025, with full-text screening and data charting scheduled for January-March 2026. Thematic synthesis and expert consultation are planned from April to May 2026. We anticipate submitting the completed scoping review paper by June or July 2026.

**Conclusions:**

This review will generate Africa’s first continent-focused evidence map of vaccine-manufacturing readiness, compiling indicators by domain, comparing frameworks, identifying gaps, and informing a multidomain Country Readiness Assessment Index for policy and investment decisions.

**Trial Registration:**

Open Science Framework 10.17605/OSF.IO/UVSWX; https://osf.io/uvswx

**International Registered Report Identifier (IRRID):**

DERR1-10.2196/81231

## Introduction

### Background

Despite substantial scientific contributions of African researchers in vaccine research and development (eg, HIV Vaccine Trials Network/South African AIDS Vaccine Initiative for HIV, South African Tuberculosis Vaccine Initiative–led tuberculosis trials, and pivotal COVID-19 vaccine studies in South Africa), local vaccine manufacturing capacity across Africa remains minimal [1], currently supplying less than 1% of the continent’s needs [2]. This heavy reliance on imports was starkly highlighted during the COVID-19 pandemic, when African countries faced delayed access to vaccines due to external supply constraints [2,3]. In response, the African Union and Africa Centres for Disease Control and Prevention (Africa CDC) have set an ambitious goal: by 2040, 60% of vaccines needed in Africa should be produced locally [1]. Achieving this target is critical for health security and equity, as it would reduce dependence on global supply chains and improve the continent’s ability to respond to epidemics.

Country-level experiences reveal significant variation in vaccine manufacturing “readiness.” South Africa, through firms like Biovac and Aspen Pharmacare, has demonstrated higher readiness in several domains, including regulatory maturity, fill-finish capacity, and active technology transfer partnerships [2,3]. Senegal’s Institut Pasteur de Dakar highlights both legacy strengths and persistent gaps, combining long-standing yellow fever vaccine production capability with newer platform investments and ongoing challenges in supply chain resilience and financing [3,4]. More recently, countries like Rwanda and Ghana have launched national vaccine manufacturing strategies; yet, they remain in the early stages of building infrastructure, policy frameworks, and regulatory systems necessary to translate political commitment into operational capacity [5,6].

These examples underscore the diversity of readiness across African countries. However, despite growing momentum and multiple global initiatives and frameworks (eg, the Partnership for African Vaccine Manufacturing [PAVM] Framework led by Africa CDC to boost Africa’s vaccine manufacturing ecosystem, World Health Organization’s (WHO) Regional Framework for strengthening local production [7], Gavi, the Vaccine Alliance African Vaccine Manufacturing Accelerator platform [8]), there is no standardized, multidomain tool for assessing a country’s vaccine manufacturing readiness. That is, no consolidated framework or index exists that integrates indicators spanning technical infrastructure, policy environment, regulatory maturity, supply chain, workforce, and financing systems [9].

### Literature Gaps

To address this gap, we conducted a search between May and August 2025 across PubMed, major gray literature repositories, Open Science Framework, and International Prospective Register of Systematic Reviews for completed or ongoing evidence syntheses on this topic (Multimedia Appendix 1). No published or registered scoping or systematic review with an equivalent scope was identified. Recently, although reports and commentaries have begun to catalog facilities, map pipelines, or describe enabling factors for vaccine production in Africa, the existing literature falls short. First, most outputs are single-domain (eg, regulatory benchmarking, financing mechanisms, or facility counts) and do not integrate across technical, regulatory, workforce, policy, and supply chain dimensions to yield a country- or a regional-level picture of “readiness.” Second, indicators are heterogeneous and inconsistently defined across sources, limiting comparability and precluding aggregation into a standardized index. Third, many assessments are cross-sectional (often COVID-19 era snapshots), providing little longitudinal insight into how readiness evolves. Fourth, several influential sources are gray literature or program documents with unclear methods, limited transparency on indicator selection or weighting, and minimal external validation. Fifth, most frameworks are not tailored to the African policy environment (eg, procurement modalities, regional market-shaping initiatives, and technology-transfer pathways), reducing their applicability. Finally, few publications explicitly link measured domains to actionable thresholds (what constitutes “ready enough”) [3,4,10,11]. These limitations underscore the need for a scoping review to systematically map concepts, indicators, and gaps across sources, and to inform the development of a transparent, multidomain Country Readiness Assessment Index (CRAI) suitable for African contexts.

### Rationale of the Scoping Review

A scoping review is therefore warranted for 3 key reasons. First, the concept of vaccine manufacturing “readiness” is multidimensional and inconsistently defined across sources ranging from regulatory maturity to workforce capacity, financing, and policy frameworks [3,4]. Scoping reviews are particularly suited to clarifying key concepts, mapping the breadth of available evidence, and identifying gaps in complex or emerging fields. Second, the available literature is heterogeneous, spanning peer-reviewed studies, technical reports, policy documents, and organizational frameworks. Systematic reviews are designed to answer narrowly focused questions on intervention effectiveness, whereas a scoping review can accommodate this diversity of evidence and synthesize it into a coherent map [12-14]. Third, no published or registered review has yet consolidated frameworks, indicators, and policies for assessing vaccine manufacturing readiness in Africa, despite clear demand from regional bodies, such as the African Union and Africa CDC, for standardized tools [3,4]. Conducting a scoping review will therefore establish the evidence base required to guide the development of a tailored readiness index and inform policy and investment priorities.

### Definition of Readiness

For the purposes of this review, readiness is defined as the extent to which a country has the foundational systems and conditions to initiate or scale local vaccine production [15]. These include regulatory capacity, enabling policies, physical infrastructure, supply chain systems, financing mechanisms, and skilled workforce availability. This review will focus specifically on readiness, as this concept is both amenable to scoping review methodology and grounded in accessible evidence sources, such as policy documents, technical reports, and regulatory tools. In contrast, related concepts like capability (operational performance) and viability (economic sustainability) rely on proprietary or facility-level data that are not consistently available [16,17].

Scoping reviews are designed to clarify concepts, collate heterogeneous evidence, and identify gaps rather than to assess operational performance or economic feasibility. Accordingly, focusing on readiness enables the review to generate a comprehensive knowledge map that is both methodologically sound and directly relevant for informing policy and investment priorities in Africa [18].

The findings from the scoping review will directly inform the subsequent development of a CRAI for vaccine manufacturing in Africa. By systematically mapping the dimensions of readiness described across peer-reviewed studies, organizational frameworks, and policy documents, the review will identify common domains, indicators, and measurement approaches that can serve as building blocks for the CRAI. Gaps revealed through the review will highlight where new indicators or methodological innovations are required. In this way, the scoping review functions not only as a synthesis of existing knowledge but also as a methodological foundation for designing and validating a tailored, multidomain tool to evaluate readiness at the country or regional level.

### Scope and Unit of Analysis

Our unit of analysis is the country, not the industry. Vaccine manufacturers operate within and are constrained by the political, regulatory, and socioeconomic context of their host countries; therefore, durable manufacturing capacity depends on country-level readiness. Assessing readiness at this level enables governments to identify priority gaps, target interventions, build capabilities, attract strategic investment, and foster international collaboration, laying the groundwork for vaccine self-sufficiency and stronger public health resilience by 2040.

Ultimately, CRAI responds to the urgent need for evidence-informed strategies to build Africa’s vaccine production capacity, will serve as a tool for benchmarking, strategic planning, and resource mobilization to support Africa’s vaccine manufacturing ambitions.

### Review Objectives

The objectives of this scoping review protocol are (1) to describe a methodological approach that will be used to review existing literature to identify, map, and synthesize the existing evidence on all relevant frameworks, indicator sets, and policies related to readiness for local human vaccine manufacturing in African countries; (2) derive and validate a taxonomy of readiness domains and indicator set from included sources; and (3) produce standardized outputs including a framework-mapping table, theme×frequency summary, and a master indicator catalog to inform construction of a CRAI (Multimedia Appendix 2).

In alignment with the Joanna Briggs Institute (JBI) scoping review methodology, we aim to broadly explore all relevant concepts and evidence on this topic [13,14,18,19]. We adopted the JBI enhancement for conduct because it provides operational, step-by-step guidance tailored to scoping reviews covering Population, Concept, and Context (PCC) framing, eligibility specification, search or selection, standardized data charting, and stakeholder consultation, thereby supporting methodological consistency across heterogeneous evidence. Specifically, the review will chart the characteristics of any published materials that define or assess a country’s readiness to produce vaccines locally. This includes global-level frameworks or readiness indices proposed by organizations such as WHO, Gavi, Coalition for Epidemic Preparedness Innovations, as well as national strategies, policy documents, and academic studies from or about specific African countries. By mapping these sources, the review seeks to understand the dimensions commonly considered part of “manufacturing readiness” (regulatory readiness, technical infrastructure, workforce, financing, supply chain, etc), and to summarize how readiness has been measured or conceptualized to date. Ultimately, the objective is to produce a comprehensive overview that will inform the development of a new composite readiness index for vaccine manufacturing in Africa.

## Methods

### Conceptual Framework

We will adapt and use a vaccine-specific taxonomy, which is Political, Economic, Social, Technological, Legal, Environmental, plus Market (informed by Political, Economic, Social, Technological, Legal, Environmental [20] model), to structure data charting and synthesis. Each domain is defined for human vaccine manufacturing readiness, with illustrative indicator examples (used only as guides; final indicators will be those extracted from sources. The indicative domains include Policy and governance, Economic and financing, Social and workforce, Technology and infrastructure (including drug substance and drug product functions, Legal: intellectual property and regulatory systems, Environmental and utilities (energy, water, and waste), and Market and demand. Examples include presence of a national strategy or roadmap (Policy and governance), viability-gap funding or guarantees (Economic and financing), Good Manufacturing Practice–trained workforce capacity (Social and workforce), drug substance bioreactor availability (Technology and infrastructure), Good Manufacturing Practice and Quality Assurance statutes (Legal), National Regulatory Authority maturity / African Vaccine Regulatory Forum participation or WHO Prequalification track record (intellectual property and regulatory systems), stable power and effluent management (Environmental and utilities), and multiyear offtake commitments (Market and demand).

### Study Design

This scoping review was developed based on the Arksey and O’Malley scoping review methodology and further informed by guidance from the JBI [14,18,19]. The JBI’s enhanced framework expands the 6 stages of Arksey and O’Malley into nine distinct stages for undertaking a scoping review: (1) defining the review question, (2) developing the inclusion and exclusion criteria, (3) describing the search strategy, (4) searching for the evidence, (5) selecting the evidence, (6) extracting the evidence, (7) charting the evidence, (8) summarizing and reporting the evidence, and (9) consulting with relevant stakeholders. For details, see Multimedia Appendix 3 on operationalization of JBI steps for vaccine-manufacturing readiness.

### Step 1: Defining the Review Question

The overall main research question was defined as “What frameworks, indicators, or policies have been documented that relate to a country’s or regional readiness for local vaccine manufacturing in African countries?” This broad question will be addressed by mapping evidence across various sources. In addition, we will consider the subquestions listed in Textbox 1 to ensure comprehensive coverage of the topic.

**Review subquestions.**

**Subquestions**

- Which frameworks or models (conceptual or analytical) have been proposed to assess or guide vaccine manufacturing capacity or readiness, low- and middle-income countries, or in Africa?
- What indicators or indices have been used to measure readiness or enabling factors for local vaccine production (eg, multifactor readiness indices, assessment checklists, and benchmarking tools)?
- What policies, strategic plans, or initiatives have African countries implemented to enhance their readiness for vaccine manufacturing, and what do these documents identify as key readiness criteria or challenges?
- How do initiatives (by the World Health Organization, Gavi, Coalition for Epidemic Preparedness Innovations, etc) define or support “readiness” for vaccine manufacturing, and how are these frameworks being applied or adapted in African contexts?
- What gaps exist in the current literature and frameworks regarding vaccine manufacturing readiness, especially in areas such as regulatory systems, technology transfer, supply chains, financing, human capital, or partnerships?
- Are there lessons learned or failed attempts at vaccine manufacturing in Africa?

These questions collectively aim to capture both the content (the existing indicators, frameworks, and policies) and the context (who developed them, for what purpose, and applicability to Africa). The scoping review approach, with its broad question, is appropriate here since our goal is to map concepts and evidence rather than to answer a narrow effectiveness question [13,14]. As recommended for scoping reviews, we maintain a broad scope but with clearly articulated key concepts (PCC) to guide the search and selection of evidence [13,14,18]. The resulting review will address these questions above and highlight areas where evidence is abundant or lacking.

### Step 2: Identifying the Relevant Studies (Defining the Inclusion and Exclusion Criteria)

To identify eligible studies and gray literature for review, we will apply the eligibility criteria outlined in Table 1, following JBI guidance on clearly specified inclusion criteria for scoping reviews [13,14,18]. Gray literature characteristics will be captured with Authority, Accuracy, Coverage, Objectivity, Date, and Significance descriptors [21].

We will apply the JBI’s PCC framework to define and identify the inclusion criteria for this scoping review [13,14,18]. The PCC elements are outlined in Table 1.

**Table 1:** Elements of the PCC (Population, Concept, and Context) framework.

Criterion	Include	Exclude
Population	Literature on any of the 55 African Union member states, multicountry or continent-wide African analyses, and global studies that report African data.	Studies with no African country data
Concept	Frameworks, indices, indicator sets, and policies that define or measure readiness for human vaccine manufacturing (infrastructure, regulatory, workforce, supply chain, finance, and markets). We defined the concept of readiness as the structural and system preconditions to initiate or scale human vaccine manufacturing. We will include items on capability or viability only when they explicitly define or operationalize readiness or contain readiness-measurable indicators.	Generic pharmaceutical and medicines manufacturing capacity papers, unless they contain vaccine-specific indicators. For example, papers focused solely on nonvaccine medicines (small-molecule active pharmaceutical agents, oral solids, and injectables unrelated to vaccines), medical devices, or diagnostics, with no vaccine-specific indicators.
Context	National or regional African initiatives; global guidance explicitly referencing Africa’s manufacturing goals.	Veterinary-only production and manufacturing readiness in other continents (unless used purely for comparison).
Publication type	Peer-reviewed articles, government, agency, international governmental organization reports; strategy documents; theses; conference papers, registered preprints, and key repositories (eg, SABINET, institutional repositories, or ministries’ websites).	Opinion or commentary without a framework or indicators, veterinary-only, non-African with no Africa application, basic research and development with no readiness link.
Language	English, French, Arabic, Portuguese (title and abstract screening used machine translation [DeepL/Google] for non-English records to enable first-pass triage.	All other languages.
Time frame	Publication years from January 1, 2010, to December 31, 2025. Pre- and post–COVID-19 era surge and policy momentum.	Publications before 2010.
Setting	Human vaccine manufacturing across the full value chain (antigen to fill-finish).	Downstream procurement, logistics, and service-delivery topics.

### Steps 3 And 4: Describing the Search Strategy and Searching the Evidence—Search Strategy

Following the JBI-recommended methodology, we will implement a three-step search strategy to identify both published and gray literature sources [13,14]. The search strategy will be developed with the guidance of a reference librarian and adapted for other databases using appropriate controlled vocabulary and syntax. The search strategy will be designed to be comprehensive, aiming to capture the full scope of literature on our topic within practical limits [13]. The planned steps are listed below

#### Limited Search

First, we will conduct an initial exploratory search in 2 or more relevant databases, such as MEDLINE (via PubMed), using preliminary keywords related to vaccine manufacturing and readiness. An example of our initial search string in PubMed might include terms such as “vaccine manufacturing” OR “vaccine production” AND readiness OR capacity OR capability OR preparedness OR “local production” AND Africa OR African OR specific country names. We will examine the titles, abstracts, and index terms of the results from this initial search to refine our understanding of how relevant literature is described. This will help identify additional keywords, synonyms, and subject headings. For instance, we might discover terms like “vaccine industrialization,” “pharmaceutical manufacturing capacity,” or specific program names (eg, PAVM) that should be incorporated.

#### Comprehensive Search

Using the insights from Step 1, we will craft a more detailed search strategy and apply it across all selected information sources. This will include multiple bibliographic databases. We anticipate searching MEDLINE (PubMed), Scopus, and Web of Science. We will also search region-specific databases or repositories, such as Africa-wide Information, African Index Medicus, or African Journals Online, to capture research published in African journals. The database search strings will combine keywords and controlled vocabulary (eg, Medical Subject Headings terms like “Drug Manufacturing” or “Vaccines” and terms for readiness or capacity). We are developing and improving a search strategy in PubMed. For details, see Multimedia Appendix 4 to retrieve relevant literature.

We will tailor the search syntax for each database (using MeSH [Medical Subject Headings] in PubMed, etc, where appropriate). We will apply date and language limits. In addition to journal databases, we will search for gray literature systematically. This includes targeted searches of organizational websites and document repositories.

#### Reference List Search and Expert Recommendations

In Step 3, we will examine the reference lists of all included sources (backward reference checking) to identify any additional publications we might have missed. We will also use forward citation tracking on key articles or frameworks (using tools like Google Scholar’s “cited by” feature) to find more recent works that cite known references. Moreover, as part of our methodology (and aligning with expert consultation plans described later), we will ask experts in the field to suggest any seminal or unpublished materials. This could include, for example, an expert alerting us to a forthcoming government policy or a workshop report that may not be indexed online. Any such sources that meet our inclusion criteria will be pursued. Throughout the search process, we will use an iterative approach: if we identify new keywords or acronyms (eg, if we find an index called “Vaccine Manufacturing Readiness Index” hypothetically, we would add that term to our search), we will rerun searches as necessary. All search strategies (database-specific strings and a list of gray literature sources searched) will be documented fully in the final protocol and subsequent report. Our goal is to ensure the search is as comprehensive as possible within resource limits, capturing published peer-reviewed studies, relevant conference papers or theses, and policy and strategy documents from the gray literature.

### Step 5: Selection of Sources of Evidence

We will follow a transparent and rigorous selection process to screen the search results and determine the final set of sources for inclusion (see completed PRISMA-ScR (Preferred Reporting Items for Systematic Reviews and Meta-Analyses extension for Scoping Reviews) checklist, mapping each item to study sections provided in Multimedia Appendix 5). The selection will proceed in multiple stages, using a PRISMA (Preferred Reporting Items for Systematic Reviews and Meta-Analyses) flow diagram to report the process.

#### Data Harmonization and Deduplication

All records retrieved from the various searches will be imported into a reference management software (Zotero). Duplicate records will be identified and removed before screening. We will also integrate results into a systematic review management tool (such as Covidence) to facilitate screening and collaboration among reviewers. We will maintain a master indicator registry that assigns a unique ID to each distinct readiness indicator or framework. All extracted criteria will be mapped to unique IDs, and counts will be computed at the unique-indicator (unique ID) level, not per document, while preserving full source provenance. Overlaps (eg, WHO and AU citing the same criterion) will not be double-counted: exact and near-duplicate items will be merged under a single unique ID.

#### Title and Abstract Screening

Two reviewers (UAA and KJU) will independently screen the titles and abstracts of all retrieved records against the inclusion criteria. We will develop a screening guide based on our PCC criteria to ensure consistency. For example, any record not mentioning vaccines or not related to Africa will be quickly excluded at this stage. If information in the title or abstract is insufficient to determine inclusion, we will err on the side of inclusion and carry it forward to full-text screening to avoid premature exclusion. The two reviewers will label each record as “include,” “exclude,” or “unsure.” We will then compare decisions and resolve any conflicts through discussion. In cases of disagreement that are not easily resolved, a third reviewer (CAN) will be available to arbitrate. Having at least two independent reviewers throughout the selection process will minimize bias and improve reliability [13]. We will keep a log of reasons for exclusion at the full-text stage, but not at the title or abstract stage, where the volume is larger, and reasons may be generic.

#### Full-Text Screening

We will obtain abstracts and the full texts (if available) of all records that passed the title or abstract stage or were marked as “unsure.” Each full-text document will then be assessed in detail by two independent reviewers (UAA and KJU) for eligibility. We will use a predefined form to evaluate inclusion criteria on full text (confirming the population is African or relevant to Africa, the concept is vaccine manufacturing readiness, etc). Reasons for exclusion will be recorded for each excluded full-text article (common reasons might be “Not about vaccine manufacturing readiness,” “Not focused on Africa,” or “Opinion piece with no framework or data”).

#### Final Inclusion

After full-text screening, we will have the final list of sources to be included in the review. We will document the number of sources included, as well as numbers excluded at each stage, with reasons (to be presented in the PRISMA-ScR flow diagram in the eventual review report). The inclusion of multiple reviewers and clearly defined criteria will enhance the methodological rigor of the selection process, ensuring we systematically identify relevant evidence while reducing the chance of omission due to error or bias.

We also plan a calibration exercise at the start of screening: the team will pilot screen a sample of titles or abstracts (50 references) together and compare decisions to refine the application of the criteria. Similarly, a pilot of a few full-text reviews will be done to standardize interpretation. This training set will help clarify any ambiguities in the criteria and improve consistency. All these steps follow best practices for scoping reviews to ensure a robust selection of evidence [13].

### Steps 6 and 7: Extracting and Charting the Evidence—Data Extraction and Charting Process

We are creating a data extraction form in Microsoft Excel that will be used to extract relevant data from all included studies. We shall first pilot the form on a variety of a few studies. Key data elements outlined in Table 2 will be extracted by UAA and KJU.

**Table 2:** Data items.

Data items	What will be captured?
Bibliographic info	Authors, year, title, source, publication type
Geographic focus	Country or region covered (eg, Kenya, multicountry, Africa-wide, and global)
Aim or purpose	Stated goal (new framework, capacity evaluation, policy analysis, etc)
Conceptual framework	Named model or pillars, or components of readiness, if provided
Indicators or criteria	Specific readiness measures listed (eg, National Regulatory Authority maturity, and workforce size)
Composite index	Existence, construction, and any scores or rankings of a readiness index
Key findings	Main facilitators or barriers, or conclusions relevant to readiness
Contextual factors	Noted enablers or constraints (tech transfer, raw-material supply, etc)
Recommendations	Actions or policy steps the source proposes to improve readiness
Stakeholder roles	Governments, industry, donors, or others identified, and their roles
Evidence type	Methodology (review, case study, survey, expert consultation, etc)

#### Data Items to Extract

For each included source (whether a journal article, report, or policy document), we plan to chart details listed in Table 2 (with adjustments as needed by source type).

#### Data Charting, Calibration, and Interrater Agreement

Two reviewers (UAA and KJU) will independently chart each source using a piloted form, reconcile by consensus, and monitor reliability (Cohen κ≥0.70). Charting will be iterative and reflexive: any field changes will be logged and applied retroactively to all previously charted sources. All data will be kept in a single versioned database. Before formal screening, 2 reviewers (UAA and KJU) will pilot the criteria on 50 titles or abstracts and 30-40 full texts. We will measure interrater reliability using Cohen kappa and percent agreement, aiming for κ≥0.70 at the title or abstract and κ≥0.75 at the full text. If thresholds are not met, we will refine the criteria and recalibrate. Disagreements will be resolved by consensus with a third reviewer (CAN) arbitration. All decisions and updates will be logged.

### Step 8: Collating, Summarizing, and Reporting the Evidence—Analysis and Synthesis of Results

The extracted data will be analyzed using descriptive and thematic synthesis techniques appropriate for scoping reviews. Our approach will align with the JBI guidance to both quantitatively summarize the characteristics of included sources and qualitatively synthesize the emergent themes. The analysis will proceed in the following manner:

1. First, we will synthesize the evidence using a hybrid deductive–inductive thematic approach. We begin with an a priori codebook derived from a readiness taxonomy (PESTLE domains, regulatory-maturity elements, and vaccine manufacturing functions such as research and development, drug substance, and fill-finish). Two reviewers (UAA and KJU) will pilot code approximately 10% of sources to develop the codebook, resolve discrepancies, and finalize code definitions and inclusion and exclusion rules.
2. Descriptive (quantitative) summary: we will produce a numerical summary of the included evidence. This will include counts and frequencies, such as the number of sources by publication type (eg, X peer-reviewed articles, Y policy papers, and Z reports), by year of publication (to see trends over time), and by country focus (how many sources focused on South Africa, how many were multi-country, etc). We will likely present a table or a chart of the distribution of sources across countries and across source types. We will also enumerate the distinct frameworks or indices identified. For example, if we find 3 major global frameworks and, say, 5 country-specific frameworks, we will note that. Additionally, we might count how many sources mention certain domains of readiness, for example, if 90% of sources mention “regulatory capacity” as important, whereas only 50% mention “environmental factors,” that is an insight. This quantitative mapping provides a landscape view of the literature.
3. Thematic analysis (qualitative synthesis): we will conduct a content analysis of the data we charted to identify key themes and categories pertaining to vaccine manufacturing readiness. Using an inductive approach, the review team will examine the extracted data (especially the framework components, indicators, and recommendations from each source) and group similar items together. Likely themes (to be confirmed by the data) might include Policy and governance (eg, presence of national vaccine manufacturing plans, political economy drivers, partnerships, political will, and regulatory frameworks), Infrastructure and Technology (eg, existing manufacturing facilities, research and development labs, and cold chain infrastructure), Human Capital (skilled workforce availability and training programs), Regulatory Systems (strength of National Regulatory Authority and regulatory pathways for licensure), Financing and Investment (availability of funding, incentives for manufacturers, and public-private partnerships), Supply Chain and Inputs (access to raw materials, supply of equipment and consumables), Demand and Market (guaranteed demand via procurement, market size, and regional purchasing pools), Partnerships and External Support (tech transfer agreements, international support like from WHO or donor funding), and other contextual factors (like public acceptance, geopolitical stability, etc, if mentioned). We will not impose these categories a priori but will derive them from repeated patterns in the data (although they are informed by known frameworks like PESTLE). For each theme, we will collate what the literature says: for example, under Regulatory Systems, we might summarize that “Many sources emphasized the importance of a mature regulatory authority for vaccine licensure; however, only a few African countries have attained WHO Level 3 regulatory maturity [22,23], indicating a gap in readiness.” Under Financing, we might note how various initiatives propose innovative financing or that Gavi/African Development Bank investments are crucial. We will also identify any specific indicators that fall under each theme, to potentially create a master list of indicators grouped by domain. This qualitative synthesis will allow us to present a narrative of what readiness entails according to existing evidence, and where emphases differ. For example, global frameworks might stress regulatory and tech transfer, while national policies might also stress workforce training or infrastructure building.
4. Comparison and gaps: part of our analysis will involve comparing frameworks and sources to see where they converge or diverge. If multiple frameworks exist, we will compare their components side by side. We will highlight any gaps in the literature, areas that are important for readiness but are not well covered. For example, if we find abundant information on technical capacity but very little on community acceptance or on environmental considerations for manufacturing, we will note that as a knowledge gap. Another gap might be the lack of any comprehensive index that aggregates these factors quantitatively, reinforcing the rationale to develop one. We will also identify if there are any best practices or success factors repeatedly cited (eg, “strong government commitment” comes up in nearly all sources, indicating a consensus that political support is key) [1].
5. Use of consultation input: if, during our expert consultation (described below), we receive additional input (eg, experts pointing out missing factors or newly released documents), we will incorporate that into the analysis. For instance, an expert might inform us about an ongoing WHO initiative to create a manufacturing readiness index, and we would then include whatever information is available about that and consider it in our synthesis, even if it was not in published literature yet.
6. The synthesis will be presented both narratively and with the aid of tables or figures. We anticipate creating:
  - A summary table of identified frameworks/indices, listing their origin, purpose, and components.
  - A figure or schematic that illustrates the common domains of readiness (perhaps a conceptual diagram showing overlapping domains from different sources).
  - A PRISMA flowchart (for the selection process, as mentioned).

By collating and summarizing in this way, the scoping review will “provide a map of what evidence has been produced” on vaccine manufacturing readiness [13,14] We are not assessing the quality of evidence per se (scoping reviews typically do not exclude based on quality), but we may comment on the nature of evidence (eg, noting if most of it is commentary vs data-driven). This comprehensive synthesis will directly inform identifying which factors to include in a new readiness index and where to focus further primary research (such as expert interviews to probe gaps). All analysis steps will be documented to ensure transparency in how we moved from raw data to our conclusions.

### Step 9: Expert Consultation and Validation

#### Overview

Consulting with content experts and stakeholders is an optional but highly valuable stage in scoping review methodology [19,24]. We have planned an expert consultation component as part of this review to enhance its validity and relevance. The consultation serves 2 main purposes: (1) to ensure our search and included sources are comprehensive, and (2) to help interpret and validate the findings, especially in terms of practical significance for policy and index development.

#### Timing and Participants

We intend to engage experts after an initial draft of our evidence map is prepared (ie, after we have preliminary results from the literature). The experts will include individuals who are deeply involved in vaccine manufacturing initiatives or policy in Africa and globally. Potential participants are officials from organizations like Africa CDC’s vaccine manufacturing programs, WHO and Gavi representatives involved in local production, leaders of vaccine manufacturing companies or institutes in Africa (eg, Institut Pasteur Dakar, Biovac in South Africa, and the new Ghana Vaccine Institute), and researchers who have published on pharmaceutical manufacturing capacity. We may also include representatives from funding bodies (World Bank and African Development Bank) who have a perspective on readiness investments. We will use purposive sampling to survey approximately key experts (2-3 per sector), expanding via snowball sampling as needed, and will stop at thematic saturation (ie, when additional interviews yield no substantively new insights), to capture a broad range of perspectives.

#### Consultation Methods

We will use a flexible approach, possibly surveys. We will prepare a summary of our scoping review findings (eg, a short presentation or summary document highlighting identified frameworks and preliminary themes) to share with the experts in advance. During the consultation, we will seek feedback on several points:

1. Completeness of the literature: we will ask if they are aware of any important frameworks, reports, or data sources that we might have missed in our search. Experts might point us to very recent or in-press materials or even internal documents that could be relevant. If any new sources are identified, we will attempt to obtain them and consider them for inclusion post hoc (provided they meet criteria).
2. Interpretation of findings: We will present the themes and domains of readiness we have extracted and ask if these resonate with their experience. Do they agree that these are the key elements of readiness? Are there factors that the literature does not emphasize but are critical in practice?
3. Validation of framework for index development: since this scoping review feeds into developing a readiness index, we will use expert input to validate which indicators or domains should likely be included. If our literature-based framework has, say, 6 domains (policy, infrastructure, workforce, etc), we will query if these cover all critical areas. Experts might also suggest how to prioritize among them or note if any proposed indicator is not a good measure in reality. This does not change the scoping review results per se, but it adds a layer of practical validation that we will report on.
4. Feasibility and data for indicators: experts might also provide insight on how certain indicators could be measured (or whether data exists), which is useful context for the next research phase.

We will document the expert consultation process and integrate it with the scoping review in two ways. First, any new sources or gray literature identified will be added to the review if possible (with an addendum search if needed). The protocol allows for this consultation stage to inform the search as an iteration. Second, in our scoping review report, we will include a section on “Consultation Results” where we summarize the key suggestions or affirmations from the experts. For example, if experts unanimously highlight the importance of a regional pooled procurement mechanism as a readiness factor (even if only a couple of documents mentioned it), we will note that the consultation reinforced this point, adding credibility. Consultation in scoping studies is known to help inform or validate findings [11], improving the usefulness of the review for end users.

By moving beyond literature alone and involving stakeholder voices, we align with an expanded methodology for scoping reviews that values knowledge user engagement. This is especially important in a dynamic, policy-driven field like vaccine manufacturing in Africa, where some knowledge may reside in practice networks or recent initiatives not yet published. The expert consultation thus serves as a bridge between evidence and real-world application, ensuring that our review outputs are not only academically thorough but also grounded in current initiatives and expert consensus. All consultation activities will be conducted in an ethical manner, obtaining consent from participants and making it clear that their input is for the research purposes of refining our understanding. Any potential conflicts of interest (eg, an expert heavily promoting a particular index) will be managed by cross-referencing with evidence from other sources.

#### Reporting and Dissemination

##### Overview

This scoping review will be reported in accordance with the PRISMA-ScR guidelines [24]. We will ensure all the key reporting items are addressed, including a clear description of our background, objectives, eligibility criteria, search strategy (with search strings for at least one database provided), selection process, data charting methods, and results of the review. All sources of information will be properly cited, and any protocol deviations will be noted. In the scoping review paper, we will also include the statement: “The objectives, inclusion criteria, and methods for this scoping review were specified in advance and documented in a protocol,” along with a reference to this protocol.

For dissemination, there are multiple avenues:

- Academic publication: We intend to publish the scoping review in a peer-reviewed journal. We will also make the published article open access for broader reach.
- Stakeholder engagement beyond academia: to facilitate policy and practice uptake, we will prepare a 2-4 page policy brief and slide deck tailored for African stakeholders (eg, Africa CDC or PAVM, World Health Organization Regional Office for Africa [WHO AFRO], National Regulatory Authorities, National Immunization Technical Advisory Groups, Ministries of Health and Industry, regional economic communities, and relevant manufacturer associations). We will seek opportunities to present findings in Africa CDC or WHO AFRO webinars or technical convenings and at regional forums (eg, the Vaccine Alliance African Vaccine Manufacturing Accelerator platform and PAVM working groups). Where feasible, we will conduct targeted briefings with priority audiences (eg, regulatory and industrial policy units) to discuss how mapped readiness domains can inform ongoing initiatives.

Throughout dissemination, we will emphasize how this scoping review fills a crucial knowledge gap and sets the stage for evidence-based interventions. By documenting existing frameworks and indicators, we can help avoid duplication of efforts and encourage stakeholders to coalesce around best practices identified. We anticipate the review becoming a reference for groups like the African Union, WHO AFRO, or national task forces when they articulate what needs to be done to enable vaccine manufacturing, essentially providing a menu of factors and examples gleaned from the literature.

##### Measuring Dissemination Success

To assess whether dissemination reaches intended audiences and informs practice, we will track (1) bibliometrics (citations, downloads, and Altmetric attention); (2) stakeholder engagement metrics (attendance at briefings or webinars, requests for follow-up or technical assistance, distribution of the policy brief or slide deck); (3) evidence of policy or practice uptake (citations or references in AU, Africa CDC, WHO AFRO or national strategy documents, inclusion in guidance and roadmaps, and invitations to contribute to working groups); and (4) early indicators of application (eg, references to the review’s domains in country assessments or pilot use to scope a readiness index).

### Ethical Considerations

Although this scoping review does not collect primary health or patient data, the planned expert consultation component involves human participants. The expert consultation component received approval from the University of Geneva Research Ethics Committee with decision form number CUREG-2025-05-87 (application submitted May 21, 2025, and approval received September 9, 2025). Written informed consent will be obtained, and data will be anonymized and securely stored.

## Results

The protocol is registered on the Open Science Framework. The initial search strategy has been finalized, and pilot searches were completed (May-August 2025). We are currently reviewing and scoping more abstracts and literature from peer-reviewed journals and gray literature. The screening calibration set (n50) will be prepared. Dual review title or abstract screening begins December 2025, with full-text screening and data charting scheduled for January-March 2026. Thematic synthesis and expert consultation are planned for April-May 2026. We anticipate submitting the completed scoping review manuscript in June or July 2026.

## Discussion

### Expected Findings

This scoping review will generate the first continent-focused evidence map of vaccine-manufacturing readiness in Africa. It goes beyond single-domain inventories to produce a multidomain taxonomy and master indicator catalog. The predefined outputs (framework-mapping table, theme-frequency summary, and indicator catalog) translate fragmented evidence into decision-oriented products. Methodologically, rigor is supported by PCC-based eligibility, dual-reviewer screening with calibration, descriptive mapping plus inductive thematic analysis, and a targeted expert consultation (5-10 informants) to validate domains and surface missed sources. Anticipated insights include (1) areas of consensus across domains (regulatory, infrastructure or technology, human capital, finance, inputs, demand or market, partnerships, and governance/context), (2) gaps (eg, inputs, energy reliability, environmental compliance), and (3) a shortlist of candidate pillars or indicators for a CRAI.

### Limitations

There are a few limitations to this scoping review. Article inclusion is restricted to papers published in English, French, Portuguese, and Arabic. Local language items may be missed, partly mitigated via regional portals and expert flagging. Gray literature heterogeneity limits methodological appraisal, consistent with JBI. No formal critical appraisal will be performed. The review offers a cross-sectional snapshot, and late-emerging initiatives may fall outside the window.

### Conclusions

This scoping review protocol has detailed a rigorous approach, following JBI methodology, to map the landscape of vaccine manufacturing readiness in African countries. By clearly outlining our objective, question, inclusion criteria, search methods, selection, data charting, analysis plan, and consultation strategy, we aim to ensure the review process is transparent and reproducible. The ultimate outcome will be a comprehensive knowledge synthesis that not only contributes to academic literature but also supports practical policy and strategy development toward Africa’s vaccine self-sufficiency goals [1]. The protocol and subsequent review will be valuable steps in a larger research endeavor to develop and validate a readiness index for vaccine manufacturing in Africa, guiding investments and actions in the coming years for strengthening health security on the continent.

## Appendix

Multimedia Appendix 1 Existing tools on vaccine manufacturing readiness.

Multimedia Appendix 2 Thematic synthesis output (domains, indicators, and frequency across sources).

Multimedia Appendix 3 Operationalization of JBI steps for vaccine-manufacturing readiness.

Multimedia Appendix 4 Search strategy.

Multimedia Appendix 5 PRISMA‑ScR flow diagram.

## Abbreviations

Africa CDC: Africa Centres for Disease Control and Prevention
CRAI: Country Readiness Assessment Index
JBI: Joanna Briggs Institute
MeSH: Medical Subject Headings
PAVM: Partnerships for African Vaccine Manufacturing
PCC: Population, Concept, and Context
PRISMA: Preferred Reporting Items for Systematic Reviews and Meta-Analyses
PRISMA-ScR: Preferred Reporting Items for Systematic Reviews and Meta-Analyses extension for Scoping Reviews
WHO: World Health Organization
WHO AFRO: World Health Organization Regional Office for Africa

## Data Availability

Data sharing is not applicable to this article as no datasets were generated or analyzed during this study.
