# Supplementary material for: Assessing the Readiness of Local Vaccine Manufacturing in African Countries: Protocol for a Scoping Review
Source: JMIR Res Protoc. 2025 Dec 23;14:e81231. doi: 10.2196/81231 (PMC12775757; doi:10.2196/81231)
Supplement: Multimedia Appendix 1 [file resprot_v14i1e81231_app1.docx]

**Table S1.**

| **Existing tool (year)** | **Geographic scope** | **What it measures** | **Principal limitation for our purpose** |
| --- | --- | --- | --- |
| **WHO Global Benchmarking Tool (GBT)** – Revision VI, 2021 | Global | Regulatory-system maturity (Maturity Levels 1-4) | Covers only the regulatory pillar; says nothing about financing, demand, or workforce |
| **Africa CDC / CHAI / PATH “Supply & Demand Mapping”** – 2023-24 | Africa | Physical fill-finish and (to a lesser extent) drug-substance capacity | One-off inventory; no governance, market, or resilience indicators |
| **Economist Impact Immunisation Readiness Index** – 2023 | 30 countries (5 African) | Enabling environment for vaccine delivery (planning, procurement, outreach, etc.) | Focuses on service-delivery; does not score manufacturing ecosystems |
| **UNIDO GMP Road-map baseline assessments** – 2019– | 19 sub-Saharan countries | Company-level compliance with GMP and business-process gaps | Micro-level, medicine-oriented; not an aggregated country index |
| **Global Health Security Index (GHSI)** – 2021 | 195 countries | 171 indicators across six preparedness categories | Only two sub-indicators (M4 & P6) touch tangentially on local vaccine production; no scoring of market viability |
| **PLOS Global Public Health cross-sectional survey** – 2023 | Global | Counted countries that already manufacture vaccines | Descriptive snapshot; no forward-looking readiness scale |
| **PAHO “Vaccine self-sufficiency” framework** – 1994 | Latin America | Qualitative checklist for national producers | Historical, region-specific, not updated or validated for Africa |
