# Supplementary material for: Assessing the Readiness of Local Vaccine Manufacturing in African Countries: Protocol for a Scoping Review
Source: JMIR Res Protoc. 2025 Dec 23;14:e81231. doi: 10.2196/81231 (PMC12775757; doi:10.2196/81231)
Supplement: Multimedia Appendix 2 [file resprot_v14i1e81231_app2.docx]

**Table S1.**

| **Theme / Domain** | **Concise definition** | **Representative indicators (examples)** | **Evidence frequency*** | **Typical source types**** | **Illustrative quote (short)** |
| --- | --- | --- | --- | --- | --- |
| Policy & Governance | National direction and oversight for vaccine manufacturing | National strategy; inter-ministerial taskforce; IP/tech-transfer policy | **k/N** ( **%** ) | Gov’t policy, WB/AfDB, peer-review | “Government leadership was decisive in…” |
| Regulatory Systems | Maturity and efficiency of NRA pathways | WHO GBT level; GMP inspections; lot-release timelines | **k/N** ( **%** ) | WHO/NRAs, peer-review | “Licensure timelines under 180 days…” |
| Infrastructure & Technology | Facilities and enabling technologies | Fill-finish capacity; QC labs; cold chain for bulk inputs | **k/N** ( **%** ) | Industry reports, gov’t | “Existing fill-finish lines enabled…” |
| Human Capital | Skilled workforce availability & pipelines | # trained QPs; bioprocess engineers; GMP training | **k/N** ( **%** ) | Academic, donor TA | “Chronic skills gaps in aseptic ops…” |
| Financing & Investment | Capital access & incentives | Public subsidies; blended finance; PPP frameworks | **k/N** ( **%** ) | WB/AfDB, donors, gov’t | “Viability improved with purchase guarantees…” |
| Demand & Market | Predictability and scale of demand | LTAs/APAs; pooled procurement; market size | **k/N** ( **%** ) | Gavi/UN supply, donors | “Regional pooled demand de-risked CAPEX…” |
| Partnerships & External Support | Tech-transfer and alliances | TT agreements; WHO PQ support; regional platforms | **k/N** ( **%** ) | WHO, CEPI, AUDA-NEPAD | “South–South TT accelerated start-up…” |
| Contextual / Enablers | Cross-cutting environment | Energy reliability; political stability; ESG, community acceptance | **k/N** ( **%** ) | Mixed | “Power reliability central to OEE > 80%…” |
