# Supplementary material for: Assessing the Readiness of Local Vaccine Manufacturing in African Countries: Protocol for a Scoping Review
Source: JMIR Res Protoc. 2025 Dec 23;14:e81231. doi: 10.2196/81231 (PMC12775757; doi:10.2196/81231)
Supplement: Multimedia Appendix 3 [file resprot_v14i1e81231_app3.docx]

**Operationalization of JBI steps for Africa vaccine-manufacturing readiness**

**Step 1. Define the review question**

- **Primary question:** What frameworks, indicators, or policies define or measure country readiness for human-vaccine manufacturing in Africa?
- **Sub-questions:** domains; operational indicators/thresholds; AU REC variation; gaps (e.g., inputs/utilities, pooled procurement).

**Step 2. Specify eligibility with PCC & decision rules**

- **Population:** 55 AU Member States; global sources only if Africa-applied or containing Africa-specific data.
- **Concept:** Readiness for human-vaccine manufacturing (regulatory maturity; policy; technology/infrastructure; workforce; inputs/utilities; financing; market/demand).
- **Context:** African national/regional initiatives or global frameworks used in Africa.
- **Include:** Peer-reviewed; Government/IGO (WHO AFRO, Africa CDC, NRAs); Gavi/CEPI/PATH/UNIDO; theses; conference papers.
- **Exclude:** Veterinary-only; generic pharma with no vaccine indicators; media/press without primary content.
- **Languages:** English, French, Portuguese, Arabic (targeted checks for Swahili/Amharic).
- **Timeframe:** 2010–2025.

**Step 3. Design the search strategy**

- **Databases:** MEDLINE (PubMed), Scopus, Web of Science, Africa-Wide Information, African Index Medicus, AJOL, SABINET.
- **Grey literature:** Africa CDC/PAVM, WHO AFRO, Gavi/AVMA, CEPI, PATH, UNIDO, AfDB, Afreximbank, UNICEF Supply Division; NRAs/ministries; AU RECs; institutional repositories; OSF/SSRN/medRxiv.
- **Key terms (examples):** vaccin*, manufactur*/production, fill finish, drug substance, bioprocess*, technology transfer, viral vector, mRNA/LNP, adjuvant, lot release, GBT maturity, pooled procurement/AVMA, inputs/utilities, customs lead times.

**Step 4. Run & document the search**

- Timestamp all runs; export full metadata; archive PDFs/HTML snapshots with access dates in OSF.
- Record de-duplication counts and search adaptations.

**Step 5. Select evidence (screening)**

- Dual screening with a written guide; calibration: 50 titles/abstracts + 10 full texts; target κ ≥ 0.70.
- Third reviewer arbitration; record full-text exclusion reasons; prepare PRISMA-ScR flow diagram.

**Step 6. Chart the data (+ Indicator Registry)**

- Extract: bibliometrics; geography/REC; framework components; indicator operationalization (definition; data source; periodicity; thresholds/weights); regulatory maturity/lot release; technology/infrastructure; workforce; financing; inputs/utilities; market/demand (AVMA/pooled procurement); partnerships/tech transfer; evidence type; provenance.
- Map criteria to a master Indicator Registry with unique IDs (UIDs); merge exact/near duplicates; parent/child links; store provenance (page/section, excerpt, archive link).

**Step 7. Collate, summarize, and report**

- Descriptive mapping by year, country/REC, and source type.
- Thematic synthesis using PESTLE‑M (Policy; Economic/financing; Social/workforce; Technology/infrastructure; Legal/IP & regulatory; Environmental/utilities; Market/demand).
- Stratify peer‑reviewed vs grey; sensitivity: peer only; exclude low‑confidence grey; parent vs child indicators.
- Visuals: framework×domain matrix; domain heatmap by REC; indicator frequency bars;

**Step 8. Expert consultation**

- After preliminary maps: ~5–10 experts (Africa CDC/PAVM, WHO AFRO, NRAs, manufacturers, financing partners).
- Purpose: identify missed sources; validate domains/indicators; feasibility insights.
- Ethics approved (University of Geneva: submitted May 21, 2025; approved Sept 9, 2025); consent; anonymized notes.

**Step 9. Reporting & data sharing**

- Report per PRISMA‑ScR; include the checklist and populated PRISMA flow.
- Share search strings, codebook, charting form, UID registry & harmonization log, and grey‑literature archive on OSF.
