# Supplementary material for: Assessing the Readiness of Local Vaccine Manufacturing in African Countries: Protocol for a Scoping Review
Source: JMIR Res Protoc. 2025 Dec 23;14:e81231. doi: 10.2196/81231 (PMC12775757; doi:10.2196/81231)
Supplement: Multimedia Appendix 4 [file resprot_v14i1e81231_app4.docx]

This search strategy will be adapted in searching other databases

("Vaccines"[Mesh] OR "Drug Industry"[Mesh] OR "Biotechnology"[Mesh] OR vaccin*[tiab])

AND

(manufactur*[tiab] OR produc*[tiab] OR biomanufactur*[tiab] OR "fill finish"[tiab] OR "fill–finish"[tiab]

OR "drug substance"[tiab] OR "drug product"[tiab] OR factory[tiab] OR plant[tiab] OR facilit*[tiab]

OR "technology transfer"[tiab] OR "tech* transfer"[tiab] OR "local produc*"[tiab])

AND

(readiness[tiab] OR preparedness[tiab] OR capability[tiab] OR capacity[tiab] OR "manufacturing readiness"[tiab]

OR "production readiness"[tiab] OR benchmark*[tiab] OR indicator*[tiab] OR metric*[tiab] OR assess*[tiab]

OR score*[tiab] OR "composite index"[tiab] OR index[tiab] OR framework[tiab] OR "maturity model"[tiab]

OR "manufacturing readiness level"[tiab] OR MRL[tiab] OR "capability maturity"[tiab] OR competency[tiab])

AND

("Africa"[Mesh] OR "Africa South of the Sahara"[Mesh] OR Africa[tiab] OR African[tiab] OR "Sub-Saharan Africa"[tiab]

OR "North Africa"[tiab] OR Maghreb[tiab] OR West Africa[tiab] OR East Africa[tiab] OR Central Africa[tiab]

OR Southern Africa[tiab] OR Algeria[tiab] OR Angola[tiab] OR Benin[tiab] ... OR Zimbabwe[tiab]

OR Afrique[tiab] OR africain*[tiab] OR "África"[tiab] OR africano*[tiab])
