# Supplementary material for: Assessing the Readiness of Local Vaccine Manufacturing in African Countries: Protocol for a Scoping Review
Source: JMIR Res Protoc. 2025 Dec 23;14:e81231. doi: 10.2196/81231 (PMC12775757; doi:10.2196/81231)
Supplement: Multimedia Appendix 5 [file resprot_v14i1e81231_app5.docx]

**PRISMA‑ScR Flow Diagram — Template**

*Preferred Reporting Items for Systematic reviews and Meta‑Analyses extension for Scoping Reviews*

| **Item** | **Count / Status** |
| --- | --- |
| **Records identified from — Databases** | (n = ) |
| **Records identified from — Registers** | (n = ) |
| **Records identified from — Websites/organizational portals** | (n = ) |
| **Records identified from — Citation searching** | (n = ) |
| **Records identified from — Other methods** | (n = ) |
| **Records after de‑duplication** | (n = ) |
| **Records screened (titles/abstracts)** | (n = ) |
| **Records excluded at T/A** | (n = ) |
| **Reports sought for retrieval** | (n = ) |
| **Reports not retrieved** | (n = ) |
| **Reports assessed for eligibility (full text)** | (n = ) |
| **Reports excluded (full text)** | (n = ) |
| **Sources of evidence charted — Peer‑reviewed** | (n = ) |
| **Sources of evidence charted — Grey literature** | (n = ) |
| **Sources included in synthesis** | (n = ) |
| **Expert consultation conducted (Yes/No; n experts = )** | (n = ) |

**Reasons for exclusion at full text**

| **Reason** | **n =** |
| --- | --- |
| **Not Africa‑specific** | (n = ) |
| **Not vaccine readiness** | (n = ) |
| **Insufficient detail** | (n = ) |
| **Other** | (n = ) |
